# Intra‐ischemic hypothermia cardioprotection involves modulation of PTEN/Akt/ERK signaling and fatty acid oxidation

**DOI:** 10.14814/phy2.15611

**Published:** 2023-02-17

**Authors:** Cody N. Justice, Xiangdong Zhu, Jing Li, J. Michael O'Donnell, Terry L. Vanden Hoek

**Affiliations:** ^1^ Center for Advanced Resuscitation Medicine, Department of Emergency Medicine University of Illinois at Chicago Chicago Illinois USA; ^2^ Department of Physiology and Biophysics University of Illinois at Chicago Chicago Illinois USA; ^3^ Center for Cardiovascular Research University of Illinois at Chicago Chicago Illinois USA

**Keywords:** fatty acid oxidation, ischemia/reperfusion, PTEN, therapeutic hypothermia

## Abstract

Therapeutic hypothermia (TH) provides cardioprotection from ischemia/reperfusion (I/R) injury. However, it remains unknown how TH regulates metabolic recovery. We tested the hypothesis that TH modulates PTEN, Akt, and ERK1/2, and improves metabolic recovery through mitigation of fatty acid oxidation and taurine release. Left ventricular function was monitored continuously in isolated rat hearts subjected to 20 min of global, no‐flow ischemia. Moderate cooling (30°C) was applied at the start of ischemia and hearts were rewarmed after 10 min of reperfusion. The effect of TH on protein phosphorylation and expression at 0 and 30 min of reperfusion was investigated by western blot analysis. Post‐ischemic cardiac metabolism was investigated by ^13^C‐NMR. TH enhanced recovery of cardiac function, reduced taurine release, and enhanced PTEN phosphorylation and expression. Phosphorylation of Akt and ERK1/2 was increased at the end of ischemia but decreased at the end of reperfusion. On NMR analysis, TH‐treated hearts displayed decreased fatty acid oxidation. Direct cardioprotection by moderate intra‐ischemic TH is associated with decreased fatty acid oxidation, reduced taurine release, enhanced PTEN phosphorylation and expression, and enhanced activation of both Akt and ERK1/2 prior to reperfusion.

## Graphical abstract

The present study reveals a new metabolic mechanism that moderate intra‐ischemic hypothermia improves cardiac function against ischemia/reperfusion injury involving the inhibition of fatty acid oxidation in an isolated heart model. Hypothermia also reduces taurine release and modulates PTEN/Akt/ERK signaling during ischemia and reperfusion.

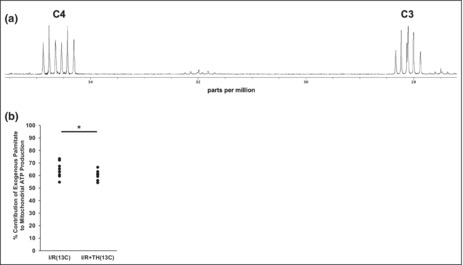

## 1 INTRODUCTION

Out‐of‐hospital sudden cardiac arrest affects approximately 350,000 adults each year in the United States, yet survival to hospital discharge remains less than 11% (Tsao et al., 2022). In cases where return of spontaneous circulation is achieved, significant mortality is attributed to a post‐cardiac arrest syndrome characterized by myocardial stunning, sepsis‐like inflammation, and hypoperfusion (Adrie et al., 2002; Bougouin & Cariou, 2013). Although two‐thirds of cardiac arrest patients with ventricular fibrillation develop hemodynamic instability requiring additional support (Oksanen et al., 2014), few therapies exist which improve post‐arrest myocardial dysfunction. Therapeutic hypothermia (TH) is potently cardioprotective in various animal models; however, clinical implementation remains challenging for the out‐of‐hospital cardiac arrest patient (Dankiewicz et al., 2021).

Cardioprotection involves activation of Akt and extracellular signal‐regulated kinase 1/2 (ERK1/2), key components of the reperfusion injury salvage kinase pathway. Several studies by our group have demonstrated a critical role for Akt in mediating protection afforded by TH (Beiser et al., 2010; Li et al., 2019; Shao et al., 2010). Akt, also known as protein kinase B, is a pro‐survival serine/threonine kinase that is expressed in nearly all cell types (Levenga et al., 2017). It is fully active when phosphorylated at both serine473 (S473) and threonine308 (T308) (Gao et al., 2005). The activity of Akt is negatively regulated by phosphatases including PTEN (Manning & Cantley, 2007). Akt enhances glucose uptake, glycolysis, and glucose oxidation through activation of GLUT‐4, phosphofructokinase‐2, and pyruvate dehydrogenase (PDH), respectively (Deprez et al., 1997; Karwi et al., 2020). Stimulation of glycolysis reduces osmotic stress and the compensatory release of amino acid taurine, possibly by mitigating the conversion of glucose to osmotically active sorbitol (Zhu et al., 2021). Furthermore, stimulation of glucose oxidation is a key mechanism for improving functional recovery, decreasing infarct size, and mitigating a pathological increase in fatty acid oxidation through reciprocal regulation according to the Randle cycle (Liu et al., 2002; Randle et al., 1963; Zuurbier et al., 2020). Additionally, Akt phosphorylates and inactivates glycogen synthase kinase 3β (GSK3β), thereby blocking mitochondrial permeability transition pore opening and subsequent apoptosis (Ajzashokouhi et al., 2023). In addition to Akt, a role for serine/threonine kinase ERK1/2 has been demonstrated in cardioprotection with mild TH (Mochizuki et al., 2013; Yang et al., 2011), although downstream protective mechanisms are less clear (Kong et al., 2019). ERK1/2 is activated by phosphorylation through the classical Ras–Raf–MEK‐ERK1/2 signaling pathway (Kong et al., 2019).

Therefore, the primary goal of the present study was to characterize the direct effect of moderate intra‐ischemic TH on PTEN/Akt/ERK signaling and metabolism in the heart. Using a Langendorff model of isolated rat heart I/R injury, we tested the hypothesis that TH modulates PTEN, Akt, and ERK1/2, and improves metabolic recovery through mitigation of fatty acid oxidation and taurine release.

## 2 MATERIALS AND METHODS

### 2.1 Ethics statement

The present investigation conforms to the eigth Edition *Guide for the Care and Use of Laboratory Animals*. All experiments were approved by the University of Illinois at Chicago Institutional Animal Care and Use Committee.

### 2.2 Langendorff isolated rat heart model

Adult male Sprague–Dawley rats were heparinized (350 USP, i.p.), anesthetized (100 mg/kg pentobarbital sodium i.p.), and hearts were excised into ice‐cold buffer for Langendorff retrograde perfusion as previously described in detail (Herr et al., 2015; O'Donnell et al., 2009; Zhu et al., 2021). Briefly, the aorta was cannulated and coronary vessels perfused at a hydrostatic pressure of 95 cm H_2_O with modified Krebs media containing (in mM): 116 NaCl, 4 KCl, 1.5 CaCl_2_, 1.2 MgSO_4_, and 1.2 NaH_2_PO_4_; equilibrated with 95% O_2_/5% CO_2_ (pH 7.4). A water‐filled latex balloon was inserted into the left ventricle with left ventricular end‐diastolic pressure (LVEDP) set to 5–10 mmHg for hemodynamic recordings (Powerlab, AD Instruments, Colorado Springs, CO). Left ventricular developed pressure (LVDP), ±dP/dt, and heart rate were recorded continuously. As an index of cardiac function, rate‐pressure product (RPP) was calculated as the product of LVDP and heart rate in beats/min.

### 2.3 Ischemia/reperfusion and hypothermia protocol

Hearts were perfused in non‐recirculating mode for 10 min followed by 10 min of perfusion in recirculating mode. Temperature was maintained at 37°C during this equilibration period. Global ischemia was induced by completely halting perfusate flow for 20 min. The intraventricular balloon was deflated during ischemia and then reinflated to the same pre‐ischemic volume 3 min after reperfusion to minimize risk of no‐reflow (Lindsey et al., 2018). For all hypothermia‐treated hearts, moderate cooling (30°C) was applied at the start of the 20‐min ischemic period and then hearts were rewarmed after 10 min of reperfusion. Cooling and rewarming were rapidly induced by rerouting the water‐jacketed organ bath to a circulating water bath pre‐equilibrated to 30°C or 37°C, respectively. Hearts in the normothermic control groups were maintained at a constant temperature of 37°C throughout the protocol. Coronary perfusate was collected from the pulmonary artery before ischemia and 10 min after reperfusion for biomarker analysis. At the conclusion of the perfusion protocol, hearts were freeze‐clamped with custom Wollenberger tongs prechilled in liquid nitrogen. Tissue was pulverized with mortar/pestle under liquid nitrogen and then stored at −80°C for subsequent analysis.

**FIGURE 1:**
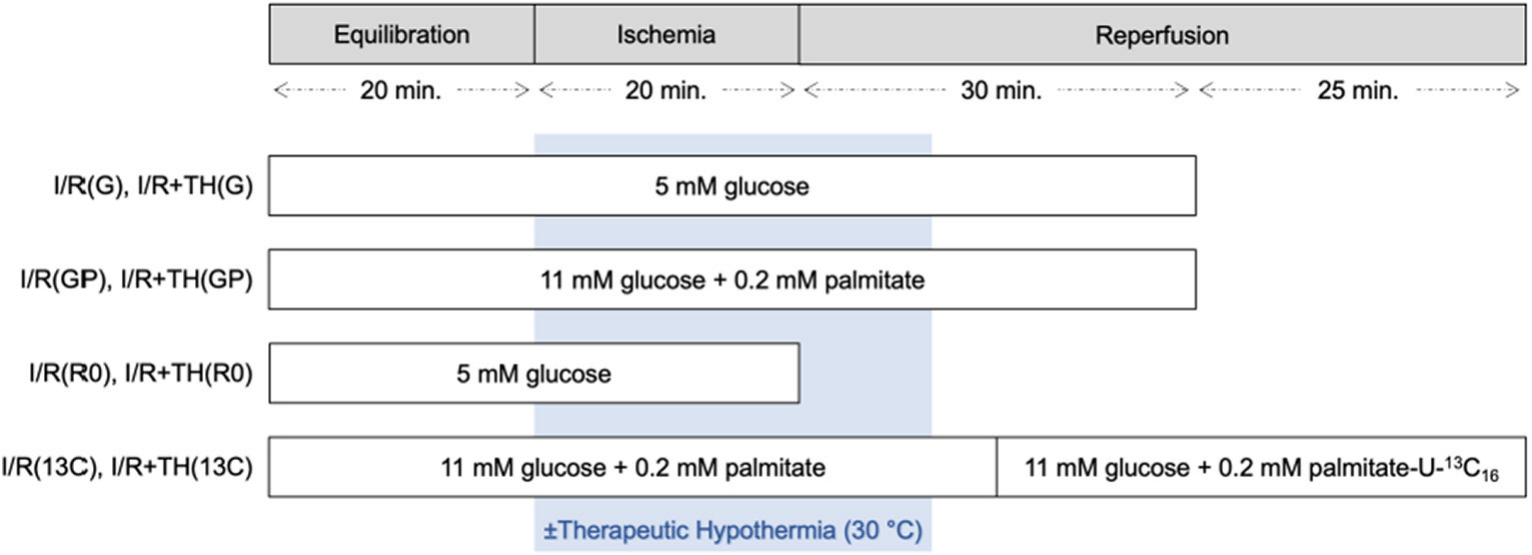
Schematic of the experimental protocol for each hypothermia intervention group and respective normothermic control group.

Figure 1 details the I/R and hypothermia protocol for all eight experimental groups. Hearts in the I/R(G) normothermic control group and I/R + TH(G) hypothermia intervention group were reperfused for 30 min following ischemia, with 5 mM glucose present throughout the entire protocol. To determine whether TH cardioprotection is affected by substrate availability, this protocol was repeated in the presence of 11 mM glucose + 0.2 mM palmitate (group I/R(GP) and group I/R + TH(GP)). Palmitate was pre‐bound to bovine serum albumin in a 3:1 molar ratio. Additional hearts were subjected to ischemia without reperfusion, with 5 mM glucose present throughout (group I/R(R0) and group I/R + TH(R0)).

### 2.4 ^13^C metabolic labeling studies

To investigate the effect of cooling protection on subsequent fatty acid metabolism, additional hearts were labeled for 40 min with media containing 11 mM glucose +0.2 mM palmitate‐U‐^13^C_16_ (Cambridge Isotope Laboratories) starting immediately after 15 min of reperfusion (group I/R(13C) and group I/R + TH(13C)) (Figure 1). Hearts in both the normothermic control group (group I/R(13C)) and hypothermia intervention group (group I/R + TH(13C)) (Figure 1) were maintained at 37°C throughout the entire ^13^C labeling period to control for any possible direct effect of temperature on enzymatic activity and labeling kinetics. Metabolites were extracted from frozen tissue by 7% HClO_4_ followed by neutralization with KOH and then lyophilization. Extracts were resuspended in D_2_O and positioned in a 14.1 T magnet for high‐resolution ^13^C‐NMR. The percent contribution of exogenous palmitate to mitochondrial ATP production was calculated by glutamate ^13^C isotopomer analysis as previously described (Malloy et al., 1990).

### 2.5 Western blot analysis

Frozen rat heart tissue was lysed in buffer containing 1% triton X‐100, 20 mM tris, 137 mM NaCl, 2 mM EDTA, 10% glycerol, 10 mM sodium pyrophosphate, 10 mM NaF, 1 mM Na_3_VO_4_, 1 mM PMSF, and 1× protease inhibitor cocktail III. Protein concentration was determined by Bradford assay (Bio‐Rad). Samples were loaded at 100 μg/lane into a 10% polyacrylamide gel for SDS‐PAGE and then transferred to a nitrocellulose membrane. Membranes were blocked for 1 h with TBST containing 5% fat‐free milk, and then incubated overnight with primary antibodies (1:1000) to pPTEN‐Ser380/Thr382/383 (#9549), PTEN (#9559), pAkt1‐S473 (#9018), Akt1 (#2938), pAkt2‐S473 (#8599), Akt2 (#2962), pAkt‐T308 (all isoforms) (#9275), pAkt‐S473 (all isoforms) (#9271), Akt (all isoforms) (#9272), pGSK3β‐S9 (#9336), pERK1/2 (#V803A), PHLPP1 (#ab135957), PP2A A Subunit (#2041), PP2A B Subunit (#2290), PP2A C Subunit (#2259), β‐tubulin (#T4026), or GAPDH (#2118). Membranes were conjugated to anti‐rabbit (#7074) or anti‐mouse (#7076) secondary antibody (1:3000) containing horseradish peroxidase for chemiluminescence. Densitometric analysis was performed using ImageJ software (NIH, Bethesda, MD, USA) with GAPDH as the loading control for each blot except for PP2A C Subunit which was normalized to β‐tubulin. All antibodies were purchased from Cell Signaling Technology, except for pERK1/2 (Promega, Madison, WI), PHLPP1 (Abcam), and β‐tubulin (MilliporeSigma).

### 2.6 Measurement of taurine and lactate

Coronary perfusate samples were analyzed by colorimetric assay per manufacturer's instruction using commercially available kits for taurine (Cell Biolabs) and lactate (Abcam).

### 2.7 Statistical analysis

Results were expressed as mean ± SD or dot plot. Between‐group comparisons for each timepoint and within‐group comparisons across timepoints were determined by two‐tailed *t* test with *p* < 0.05 considered statistically significant.

## 3 RESULTS

### 3.1 Intra‐ischemic cooling enhances recovery of myocardial function

There were no significant differences in preischemic cardiac functional parameters when comparing I/R(G) hearts to I/R + TH(G) hearts or when comparing I/R(GP) hearts to I/R + TH(GP) hearts (Table 1).

**TABLE 1:** Contractile parameters at preischemic baseline and after 30 min of reperfusion following ischemia.

	I/R(G)	I/R + TH(G)	I/R(GP)	I/R + TH(GP)
*n*	7	7	7	7
Preischemic period, 20 min				
RPP, mmHg × beats/min	29,654 ± 5317	28,224 ± 1808	24,972 ± 5259	23,591 ± 6523
LVDP, mmHg	135 ± 15	130 ± 11	104 ± 20	113 ± 8
LVEDP, mmHg	8.3 ± 1.2	7.7 ± 1.3	7.6 ± 0.7	7.1 ± 0.8
LVP, mmHg	143 ± 15	138 ± 10	112 ± 20	120 ± 8
Heart rate, beats/min	219 ± 24	219 ± 24	243 ± 19	207 ± 48
−dP/d*t*, mmHg/s	−2.79 ± 0.55	−2.58 ± 0.48	−2.04 ± 0.35	−2.00 ± 0.30
+dP/d*t*, mmHg/s	3.28 ± 1.21	3.00 ± 0.91	2.89 ± 0.76	2.80 ± 0.68
Reperfusion, 30 min				
RPP, mmHg × beats/min	18,237 ± 5882*	24,725 ± 2943* ^,^ ^#^	17,953 ± 5620*	23,064 ± 6334
LVDP, mmHg	103 ± 20*	119 ± 13	95 ± 27	112 ± 14
LVEDP, mmHg	30.9 ± 9.4*	8.7 ± 2.8^#^	30.3 ± 16.8*	4.8 ± 4.0^^^
LVP, mmHg	133 ± 19	127 ± 13	126 ± 24	116 ± 11
Heart rate, beats/min	175 ± 38*	209 ± 24	188 ± 22*	207 ± 47
−dP/d*t*, mmHg/s	−2.10 ± 0.55*	−2.40 ± 0.39	−1.93 ± 0.75	−2.09 ± 0.34
+dP/d*t*, mmHg/s	2.48 ± 0.89	2.74 ± 0.87	2.66 ± 0.94	2.77 ± 0.53

*Note*: Values are expressed as mean ± SD.

Abbreviations: dP/d*t*, change in pressure with respect to change in time; LVDP, left ventricular developed pressure; LVEDP, left ventricular end‐diastolic pressure; LVP, left ventricular pressure; *n*, number of animals; RPP, rate‐pressure product.

* *p* < 0.05, significant difference from preischemic values.

^#^ *p* < 0.05, significant difference from I/R(G) control group.

^^^ *p* < 0.05, significant difference from I/R(GP) control group.

Recovery of cardiac function was monitored for 30 min after 20 min of global ischemia in hearts perfused with 5 mM glucose (groups I/R(G) and I/R + TH(G)). RPP was reduced by 38.5% in I/R(G) control hearts following I/R injury, consistent with significant myocardial stunning (Table 1). Additionally, compared to preischemic baseline, I/R(G) hearts displayed significantly reduced LVDP, elevated LVEDP, reduced heart rate, and reduced −dP/d*t*. RPP was reduced by only 12.4% in I/R + TH(G) hearts. Post‐ischemic RPP was significantly higher in I/R + TH(G) hearts compared to I/R(G) hearts. Additionally, post‐ischemic LVEDP was significantly lower in I/R + TH(G) hearts compared to I/R(G) hearts.

To determine whether TH cardioprotection is modulated by exogenous fatty acids, these experiments were repeated with media containing a physiological mixture of 11 mM glucose +0.2 mM palmitate (groups I/R(GP) and I/R + TH(GP)). The cardioprotective effect of TH was negligibly affected by the presence of additional glucose and fatty acids as evidenced by similar patterns in functional recovery (Table 1).

### 3.2 Intra‐ischemic cooling effects on PTEN/Akt/ERK after reperfusion

**FIGURE 2:**
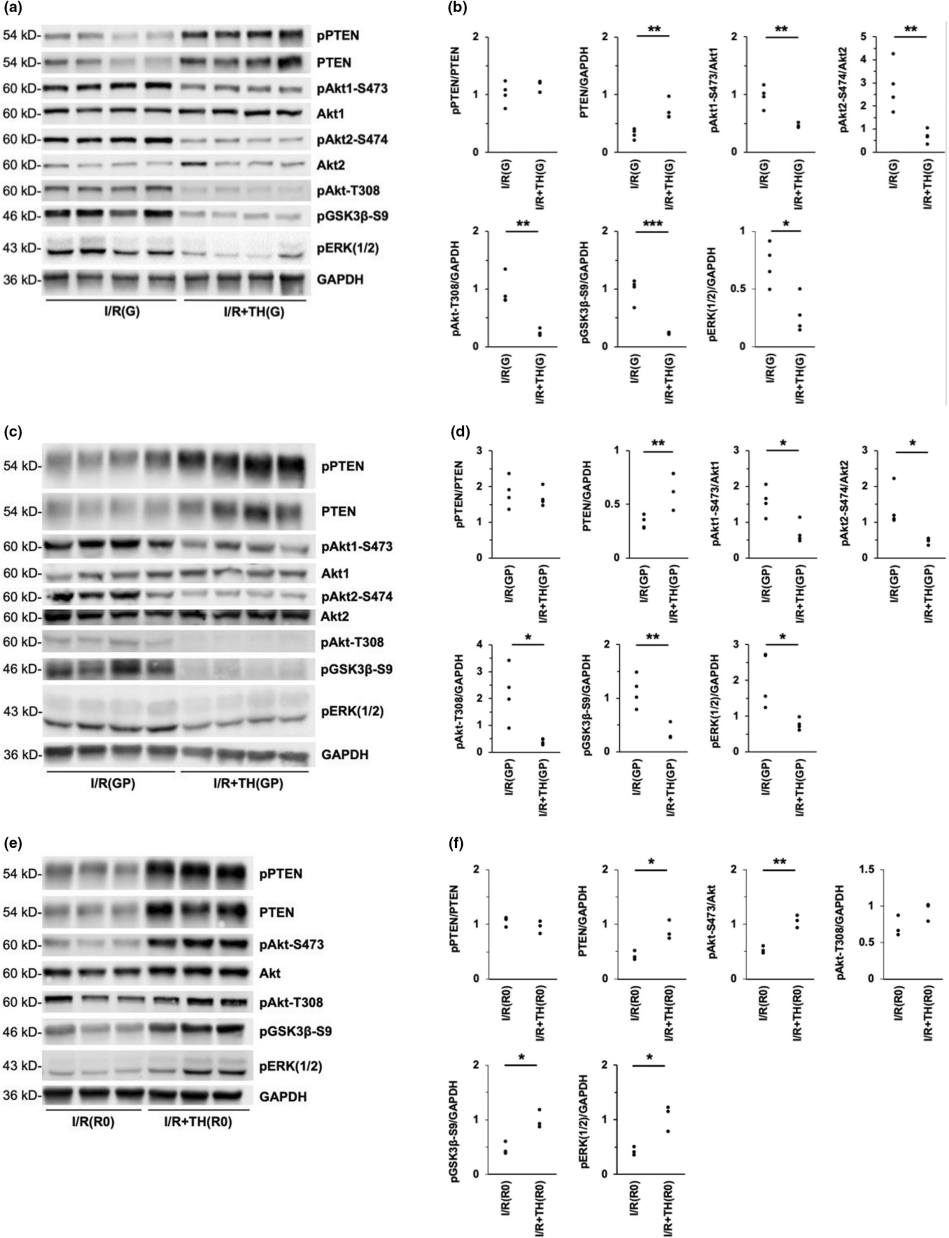
Therapeutic hypothermia enhanced PTEN expression and temporally modulated activation of Akt and ERK1/2 pathways. (a) Cardiac protein expression in hearts subjected to 20 min of ischemia and 30 min of reperfusion in the presence of 5 mM glucose (groups I/R(G) and I/R + TH(G)). (b) Densitometric analysis of protein expression in hearts from groups I/R(G) and I/R + TH(G). (c) Cardiac protein expression in hearts subjected to 20 min of ischemia and 30 min of reperfusion in the presence of 11 mM glucose +0.2 mM palmitate (groups I/R(GP) and I/R + TH(GP)). (d) Densitometric analysis of protein expression in hearts from groups I/R(GP) and I/R + TH(GP). (e) Cardiac protein expression in hearts subjected to 20 min of ischemia without reperfusion in the presence of 5 mM glucose (groups I/R(R0) and I/R + TH(R0)). (f) Densitometric analysis of protein expression in hearts from groups I/R(R0) and I/R + TH(R0). *p < 0.05, **p < 0.01, ***p < 0.001. *n* = 4 (a–d) or *n* = 3 (e, f) for each group.

Hearts were harvested after 20 min of ischemia and 30 min of reperfusion for Western blotting analysis of total protein expression and relative phosphorylation (Figure 2a–d). Compared to I/R(G) hearts, hearts in the I/R + TH(G) group displayed significantly increased total PTEN expression, while the ratio of phospho‐PTEN:total PTEN was unchanged (Figure 2a,b). Additionally, hearts in the I/R + TH(G) group displayed significantly decreased phosphorylation of Akt1‐S473, Akt2‐S474, Akt‐T308, GSK3β‐S9, and ERK1/2 (Figure 2a,b). Similar differences in protein phosphorylation were observed between groups I/R(GP) and I/R + TH(GP) (Figure 2c,d).

### 3.3 Intra‐ischemic cooling effects on PTEN/Akt/ERK prior to reperfusion

The suppressive effect of TH on post‐reperfusion Akt activation was surprising considering that functional recovery was enhanced with TH treatment. Therefore, we investigated the effect of TH on protein expression and relative phosphorylation prior to reperfusion. Hearts in groups I/R(R0) and I/R + TH(R0) were harvested for Western blotting at the end of the 20‐min ischemic period without reperfusion (Figure 2e,f). Compared to I/R(R0) hearts, hearts in the I/R + TH(R0) group displayed significantly increased phosphorylation of Akt‐S473, GSK3β‐S9, and ERK1/2 (Figure 2e,f). A trend toward increased Akt‐T308 phosphorylation was observed with TH treatment, although statistical significance was not reached (*p* = 0.11) (Figure 2e,f). Additionally, hearts in the I/R + TH(R0) group displayed significantly increased total PTEN expression compared to I/R(R0) hearts, while the ratio of phospho‐PTEN:total PTEN was unchanged (Figure 2e,f).

**FIGURE 3:**
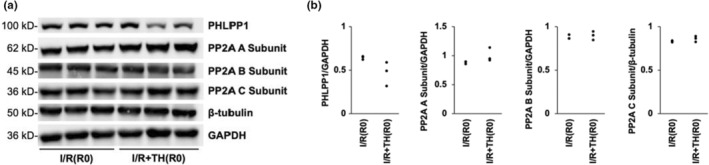
Therapeutic hypothermia was not associated with changes in expression of PHLPP1 or PP2A prior to reperfusion. (a) Cardiac protein expression in hearts subjected to 20 min of ischemia without reperfusion in the presence of 5 mM glucose (groups I/R(R0) and I/R + TH(R0)). (b) Densitometric analysis of protein expression in hearts from groups I/R(R0) and I/R + TH(R0). *n* = 3 for each group.

To determine if Akt activation was regulated by phosphatases other than PTEN, expression of PHLPP1, and PP2A was quantified in hearts from groups I/R(R0) and I/R + TH(R0) (Figure 3a,b). No differences in PHLPP1 expression or PP2A expression (including PP2A subunits A, B, and C) were observed with TH treatment (Figure 3a,b).

### 3.4 Intra‐ischemic cooling attenuates cardiac release of taurine and lactate

**FIGURE 4:**
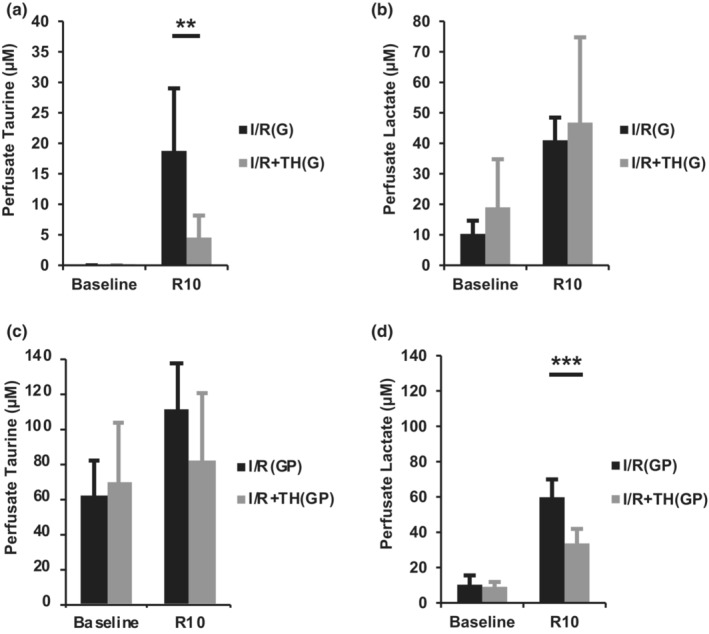
Coronary perfusate taurine and lactate concentration at preischemic baseline and after 10 min of reperfusion (R10). (a) Perfusate taurine levels from groups I/R(G) and I/R + TH(G). (b) Perfusate lactate levels from groups I/R(G) and I/R + TH(G). (c) Perfusate taurine levels from groups I/R(GP) and I/R + TH(GP). (d) Perfusate lactate levels from groups I/R(GP) and I/R + TH(GP). Values are expressed as mean ± SD; **p < 0.01, ***p < 0.001. *n* = 6 for I/R(G), *n* = 8 for I/R + TH(G), *n* = 7 for I/R(GP), *n* = 7 for I/R + TH(GP).

Considering the observation that TH modulates the response of Akt to I/R, we next investigated whether TH treatment affects cardiac release of metabolites related to Akt signaling and cardiac arrest outcomes—taurine and lactate (Donnino et al., 2014; Herzog et al., 2020; Schaffer et al., 2014). Coronary perfusate was collected before ischemia and after 10 min of reperfusion (R10) for assessment of taurine and lactate release (Figure 4a–d). In groups I/R(G) and I/R + TH(G), taurine was undetectable at preischemic baseline and no significant difference in baseline lactate was observed (Figure 4a,b). Perfusate taurine at R10 was significantly higher in I/R(G) hearts compared to I/R + TH(G) hearts (18.8 ± 10.3 μM vs. 4.6 ± 3.6 μM, respectively, *p* < 0.01) (Figure 4a). In groups I/R(GP) and I/R + TH(GP), no significant difference in baseline levels of taurine or lactate were observed (Figure 4c,d). Perfusate taurine at R10 was lower in I/R + TH(GP) hearts compared to I/R(GP) hearts, although the result did not reach statistical significance (110.7 ± 25.8 μM vs. 81.2 ± 38.7 μM, respectively, *p* = 0.12) (Figure 4c). Perfusate lactate at R10 was significantly higher in I/R(GP) hearts compared to I/R + TH(GP) hearts (59.8 ± 10.1 μM vs. 33.7 ± 8.2 μM, respectively, *p* < 0.001) (Figure 4d).

### 3.5 Intra‐ischemic cooling attenuates exogenous palmitate oxidation after reperfusion

**FIGURE 5:**
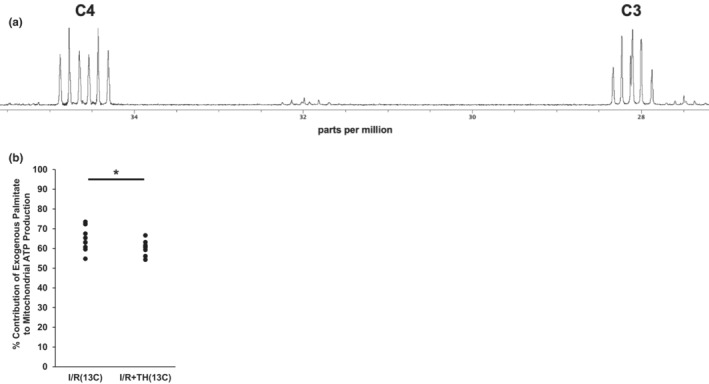
Therapeutic hypothermia treatment significantly decreased the contribution of fatty acid oxidation to total mitochondrial ATP. (a) Representative ^13^C‐NMR spectrum from a heart perfused according the experimental protocol for groups I/R(13C) and I/R + TH(13C). C3 and C4 denote resonances from glutamate carbons 3 and 4, respectively. (b) Hypothermia treatment significantly decreased the percent contribution of exogenous palmitate oxidation to total mitochondrial ATP production. *p < 0.05. *n* = 10 for I/R(13C), *n* = 9 for I/R + TH(13C).

Next, we investigated whether the cardioprotective effect of TH was associated with significant changes in metabolism, as increased fatty acid oxidation is linked with I/R myocardial stunning (Liu et al., 2002; Saddik & Lopaschuk, 1992). Hearts were perfused with 11 mM unlabeled glucose and 0.2 mM palmitate‐U‐^13^C_16_ after ischemia to assess the effect of intra‐ischemic TH on exogenous fatty acid oxidation by ^13^C‐NMR (Figure 5a). Compared to normothermic control hearts (group I/R(13C)), TH‐treated hearts (group I/R + TH(13C)) displayed significantly decreased exogenous palmitate oxidation (59.8 ± 3.9% vs. 65.2 ± 7.3%, respectively, *p* < 0.05) (Figure 5b).

To investigate changes in glucose oxidation, an additional series of experiments were conducted in which hearts were perfused with media containing 0.2 mM unlabeled palmitate +11 mM D‐glucose‐1,6‐^13^C_2_. Under these conditions, the fractional enrichment of acetyl‐CoA by glutamate isotopomer analysis fell below the lower limit of detection (data not shown).

## 4 DISCUSSION

To our knowledge, we report for the first time that moderate TH reduced fatty acid oxidation, modulated PTEN expression, and reduced taurine release following I/R injury in an isolated heart model. The use of this model is critical for testing the direct cardioprotective effect of TH irrespective of secondary whole‐body effects.

### 4.1 TH and I/R

The present study adds to a large body of evidence demonstrating the direct cardioprotective effect of TH in states of I/R injury. Relatively few studies however have examined the protective effect of mild‐to‐moderate TH using an isolated rat heart model. We found that intra‐ischemic cooling to 30°C enhanced LV functional recovery in isolated rat hearts subjected to 20 min of global, no‐flow ischemia. These results are in accordance with a study by Hansson et al. in which isolated rat hearts were cooled to 32°C during the latter half of a 30‐min ischemic insult. In that study, TH led to significantly decreased infarct size, attenuated release of lactate dehydrogenase, and enhanced recovery of RPP (Hansson et al., 2015). The protective effect of TH on infarct size and functional recovery was greater in hearts which were cooled to 26°C. Taken together, these results demonstrate that rapid application of mild‐to‐moderate intra‐ischemic TH leads to enhanced functional recovery in the non‐paced, isolated rat heart. This is supported by other studies in which TH initiated during ischemia or immediately after reperfusion led to significantly decreased LV infarct size following global ischemia in the isolated rat heart (Hjortbak et al., 2021; Mochizuki et al., 2012).

### 4.2 TH and PTEN

The present study demonstrates that moderate intra‐ischemic TH preserves PTEN expression and phosphorylation over the course of ischemia and that these changes persisted for at least 30 min after reperfusion. Using an in vivo rat stroke model, Zhao et al. demonstrated that intra‐ischemic TH (30°C) preserved phospho‐PTEN levels in the ischemic penumbra after 1 h of transient cerebral ischemia (Zhao et al., 2005). Administration of the PI3K inhibitor LY294002 significantly worsened cerebral infarct size in TH‐treated animals, indicating a critical role for PI3K in mediating the protective effects of TH in cerebral ischemia. In a similar study by Lee et al., intra‐ischemic TH (30°C) preserved levels of PTEN and phospho‐PTEN in the brain after 1 h of transient cerebral ischemia (Lee et al., 2009). Taken together, these results demonstrate that TH attenuates ischemia‐induced PTEN degradation in both the heart and brain.

The mechanism by which TH attenuates ischemia‐induced PTEN degradation may involve modulation of phosphatase activity, proteasome activity, or generation of reactive oxygen species. Upon dephosphorylation, PTEN becomes activated but is rapidly degraded (Lee et al., 2009). In the isolated rat heart, administration of okadaic acid (PP2A inhibitor) or MG‐132 (proteasome inhibitor) attenuated ischemia‐induced PTEN degradation under normothermic conditions (Cai & Semenza, 2005), raising the question of whether the TH preserves PTEN expression through inhibition of PP2A‐ or proteasome‐dependent mechanisms. TH did not affect PP2A expression in the present study, which suggests that this mechanism was not involved. Alternatively, TH may preserve PTEN expression through attenuation of reactive oxygen species. In the brain, TH attenuates the production of reactive oxygen species after stroke (Lee et al., 2009). In normothermic control animals, remaining phospho‐PTEN signal did not co‐localize with superoxide products (Lee et al., 2009). Furthermore, administration of reactive oxygen species scavenger S‐PBN significantly reduced infarct size and attenuated the decline of phospho‐PTEN in the ischemic penumbra (Lee et al., 2009). Whether these findings can be extended to the heart remains to be determined.

The importance of these findings is underscored by recent investigations demonstrating that PTEN inhibition is a promising treatment strategy for cerebral or cardiac I/R injury. Pharmacological PTEN inhibition attenuates apoptosis of hypoxic cardiomyocytes in vitro, improves isolated rat heart functional recovery after I/R, reduces infarct size after in vivo myocardial infarction, and improves survival and cardiac energetic recovery after in vivo mouse cardiac arrest (Keyes et al., 2010; Li et al., 2015; Zhu et al., 2014, 2021). Further translation of this treatment strategy is limited by the current unavailability of PTEN inhibitors which retain efficacy when administered after the onset of ischemia.

### 4.3 TH and Akt

In the present study, TH was associated with significantly enhanced Akt phosphorylation prior to reperfusion, but significantly decreased phosphorylation after 30 min of reperfusion. Inhibition of PTEN phosphatase activity by TH may explain enhanced Akt phosphorylation prior to reperfusion, considering that protein levels of PHLPP1 and PP2A were unaffected despite enhanced PTEN expression. The finding that TH suppressed Akt activation during reperfusion may be due to enhanced PTEN expression prior to reperfusion, as it was previously demonstrated that the degree of Akt activation during reperfusion depends upon the extent of ischemia‐induced PTEN degradation (Cai & Semenza, 2005). Our findings in the rat heart model are consistent with our previous work in primary cardiomyocytes subjected to 90 min of hypoxia wherein intra‐ischemic TH (32°C) enhanced Akt phosphorylation prior to reoxygenation (Shao et al., 2010). Similar trends in Akt phosphorylation were reported in brain samples from preclinical models of stroke and cerebral hypoxia (Diao et al., 2020; Lee et al., 2009; Tomimatsu et al., 2001; Zhao et al., 2005). Administration of API‐2 (Akt inhibitor) before in vitro hypoxia significantly increased cardiomyocyte cell death in TH‐treated cells but not normothermic control cells, suggesting that Akt activation during ischemia is more protective than the compensatory increase in Akt activation during reperfusion (Shao et al., 2010). Our findings support this notion, with TH‐treated hearts displaying enhanced Akt activation prior to reperfusion along with enhanced functional recovery after I/R. This time‐sensitivity may partly explain mixed results for glucose‐insulin‐potassium therapy when administered after ischemia onset for acute coronary syndrome (Selker et al., 2012).

These results diverge from preclinical in vivo cardiac arrest studies in which cooling to 30–34°C during CPR or shortly after return of spontaneous circulation were associated with enhanced post‐reperfusion Akt phosphorylation in the heart and brain (Beiser et al., 2010; Hsu et al., 2009; Jahandiez et al., 2019; Li et al., 2019). These studies did not report on PTEN expression or pre‐reperfusion Akt phosphorylation, making direct comparisons difficult. Divergent results may be explained by a host of differences between in vivo cardiac arrest models and the ex vivo rat heart, including neurohumoral response, cooling protocol, exogenous epinephrine administration, and ischemia duration. In spite of observed differences in post‐reperfusion Akt phosphorylation between models, prior investigations support the conclusion that Akt signaling plays a critical role in mediating protection afforded by TH in cardiac arrest (Beiser et al., 2010; Li et al., 2019).

### 4.4 TH and ERK

In addition to PTEN/Akt, we investigated whether TH modulates activation of ERK1/2—another key component of the reperfusion injury salvage kinase pathway. Similar to Akt, ERK1/2 phosphorylation was significantly enhanced in TH‐treated hearts prior to reperfusion, but significantly decreased phosphorylation was observed after 30 min of reperfusion. These results agree with a previous report which demonstrated that mild cooling (35°C vs. 38.5°C) attenuated the decline in ERK1/2 phosphorylation over the course of 30 min of regional ischemia in isolated rabbit hearts (Yang et al., 2011). ERK1/2 activation is generally protective in the setting of myocardial ischemia, although this is not a uniform observation across the literature (Kong et al., 2019). With respect to TH, previous studies demonstrate that pharmacological ERK1/2 inhibition abrogates TH cardioprotection in rat and rabbit models of I/R injury (Mochizuki et al., 2013; Yang et al., 2011). The mechanism by which TH regulates cardiac ERK1/2 activation remains poorly understood, but could potentially involve crosstalk with PTEN/Akt as observed in other organs and in cancer (Chetram & Hinton, 2012; Zhou et al., 2015).

### 4.5 TH and cardiac metabolism

After confirming that TH treatment improved LV functional recovery, we investigated whether this finding was correlated with alterations in cardiac metabolism as increased fatty acid oxidation is linked with I/R myocardial stunning (Liu et al., 2002; Saddik & Lopaschuk, 1992). The present study provides new insight and direct evidence that TH cardioprotection from I/R injury is associated with decreased exogenous fatty acid oxidation. While it has been known for decades that pharmacological methods to increase cardiac glucose utilization (e.g. dichloroacetate, glucose‐insulin‐potassium) or decrease fatty acid utilization (e.g. trimetazidine) are generally cardioprotective in the context of I/R (Randle et al., 1963; Zuurbier et al., 2020), very few studies have investigated the role of TH in this regard. For example, TH treatment is associated with attenuated lactate generation and increased activation of PDH in mouse models of cardiac arrest; however, direct measurements of cardiac metabolism by isotopic labeling were not conducted in prior studies (Li et al., 2019; Piao et al., 2017). All hearts in the present study were maintained at 37°C throughout the entire labeling period to control for any possible direct effect of temperature on ^13^C labeling kinetics. Therefore, the observed suppression of fatty acid oxidation by TH must be attributed to modulation of events occurring prior to the labeling period such as PDH oxidation, malonyl‐CoA depletion, buildup of toxic fatty acid intermediates, or depletion of endogenous energy stores.

### 4.6 TH and taurine release

Taurine is a conditional amino acid which is retained at high concentrations in the heart and is localized preferentially to the mitochondrion (Schaffer et al., 2014). Large amounts are exported from the heart after reversible I/R injury in order to counteract cellular osmotic stress (Schaffer et al., 2014). In the present study, we report for the first time that intra‐ischemic TH was associated with attenuated cardiac taurine release during reperfusion, indicating decreased osmotic stress. This finding is of potential clinical relevance, as it was recently reported that elevated serum taurine on admission to the hospital after out‐of‐hospital cardiac arrest was associated with increased in‐hospital mortality and poor neurological outcome (Herzog et al., 2020). Therefore, modulation of cardiac taurine release by TH should be carefully considered in future studies assessing taurine as a biomarker for cardiac arrest and ischemic heart pathologies. It remains to be determined whether reversible release of cardiac taurine leads to rapid intracellular taurine depletion in the setting of I/R, and whether cardiac function would be affected as a result.

### 4.7 Conclusion

Direct cardioprotection by moderate intra‐ischemic TH is associated with decreased fatty acid oxidation, reduced taurine release, enhanced PTEN phosphorylation and expression, and enhanced activation of both Akt and ERK1/2 prior to reperfusion.

## 5 LIMITATIONS

There are several limitations of the present study. Ex vivo isolated hearts are dennervated and therefore intrinsic heart rate deviates from in vivo values. Additionally, buffer‐perfused hearts may not recapitulate other potential in vivo factors such as the neurohumoral response to ischemia. However, this model offers unique advantages in that the direct effect of TH on the intact heart can be investigated while all other variables—such as temperature, substrate concentration, ischemia duration, and pH—are tightly controlled across experiments. Additionally, we did not investigate cardiac metabolism using additional physiological substrates such as lactate, pyruvate, and ketones; however, glucose and fatty acids represent the major sources of fuel for the beating heart (Zuurbier et al., 2020). Finally, this study is observational in nature as we did not employ pharmacological inhibitors to investigate mechanistic relationships between TH, signaling, and metabolism.

## AUTHOR CONTRIBUTIONS

1. Cody N. Justice: Experimental design, collection of data, interpretation of data, drafting of manuscript, revision of manuscript draft.
2. Xiangdong Zhu: Experimental design, interpretation of data, revision of manuscript draft.
3. Jing Li: Experimental design, interpretation of data, revision of manuscript draft.
4. J. Michael O'Donnell: Experimental design, interpretation of data, revision of manuscript draft.
5. Terry L. Vanden Hoek: Experimental design, interpretation of data, revision of manuscript draft.

All authors agree on the final version of the manuscript as written.

## FUNDING INFORMATION

This work was supported by NIH grants R01HL147031 (TVH), R01GM120485 (TVH, JMO), T32HL139439 (CNJ), and F30HL165836 (CNJ).

## CONFLICT OF INTEREST STATEMENT

The authors declare no conflict of interest.
